# Modified SIRS Criteria for Patients ≥ 65 Years with Addition of Altered Mental Status and Reduced Heart Rate for Atrioventricular Nodal Blockers

**DOI:** 10.5811/westjem.50735

**Published:** 2026-02-27

**Authors:** Lauren Gould, Eden Crowsey, Tzeferaw Sahadeo, Rita Gillespie

**Affiliations:** * Lakeland Regional Hospital, Department of Emergency Medicine, Lakeland, Florida; † Lakeland Regional Hospital, Department of Research and Sponsored Studies, Lakeland, Florida; ‡ Lakeland Regional Hospital, Data Analytics, Lakeland, Florida

## Abstract

**Introduction:**

Sepsis is a life-threatening condition caused by an exaggerated immune response to infection, causing damage to the body’s own tissues and organ dysfunction. The elderly are at higher risk for mortality from sepsis compared to younger adults. Our objective in this study was to evaluate the use of a modified systemic inflammatory response syndrome (SIRS) criteria for patients ≥ 65 years of age including new criteria of reduced heart rate (> 75 rather than 90 beats per minute [bpm]) for patients taking atrioventricular nodal blocking drugs and altered mental status.

**Methods:**

This was a retrospective observational study sampling patients ≥ 65 years of age diagnosed with sepsis. We compared our proposed modified SIRS criteria to the original criteria (heart rate, white blood cell count, respiratory rate, and temperature). Our primary outcome measure was comparing sensitivity and specificity of each model. We performed a regression analysis to evaluate the relationship of each individual criterion and its association with sepsis. Approximately half (47.1%) of the sampled population were taking an atrioventricular nodal blocking drug.

**Results:**

Based on a 1:1 case-matched dataset, the modified SIRS criteria yielded a higher sensitivity (98.9%; 95% CI, 98.4–99.2%) compared to the original criteria (97.7%; 97.0–98.2%) in diagnosing sepsis and a lower specificity (14.1%, 12.8–15.5%) compared to the original criteria (20.5%; 18.9–22.1%). The modified model demonstrated an area under the curve (AUC) of 0.797 (95% CI, 0.785–0.809; P < .001), outperforming the original model (AUC 0.764; 0.751–0.778; P < .001). Altered mental status had the second highest individual specificity for sepsis (88.4%; 87.1– 89.6%), and third was the reduced heart rate > 75 bpm for patients using atrioventricular nodal blockers criterion (53.9%; 51.9–55.8%). Among 1,164 sepsis patients receiving atrioventricular nodal blockers, 83 additional cases (7.1%; 5.8–8.8%) were identified solely by the modified heart rate ≥ 75 bpm criterion.

**Conclusion:**

The modified SIRS criteria is associated with minimally higher but statistically significant rates of identifying sepsis at the cost of reduced specificity. These new criteria identify an additional 1.21% of septic patients in the vulnerable elderly population with a 6.4% reduction in specificity. Overall, sensitivity increased marginally at the expense of specificity with the modified criteria. However, the new criteria of altered mental status and 75bpm for patients taking atrioventricular nodal blocking medications had the second and third highest individual specificity for sepsis, respectively.

## INTRODUCTION

Sepsis is a life-threatening condition that occurs when the body has an exaggerated immune response to infection causing damage to the body’s own tissues and organ dysfunction.^1^ Sepsis is a global burden with 48.9 million annually documented cases worldwide and 11 million sepsis-related deaths, representing 20% of deaths globally each year.^2^,^3^ At least 1.7 million adults develop sepsis annually in the United States, and at least 350,000 adults who develop sepsis die during hospitalization or are discharged to hospice.^4^ Sepsis is the number one cause of death in hospitalized patients and is the greatest cost burden of hospitalization in the U.S., accounting for > $53 billion of in-hospital costs each year.^5^ The elderly population is at much higher risk from sepsis with a mortality rate 1.3–1.5 times higher than that of younger adults. Adults ≥ 65 years of age are 13 times more likely to be hospitalized with sepsis compared with younger patients. Elderly patients ≥ 85 of age have mortality rates five times higher than those 65–74.^6^,^7^

Early recognition and treatment of sepsis is vital to increase a patient’s survivability. The mortality risk from sepsis increases by approximately 7–9% each hour that treatment is delayed.^8^,^9^ The systemic inflammatory response syndrome (SIRS) criteria is a tool used to identify sepsis early and initiate treatment as soon as possible. The SIRS criteria include white blood cell count, heart rate, respiratory rate, and temperature (Table 1). Two or more positive criteria in addition to an identified source of infection is considered a positive diagnosis for sepsis.

The SIRS criteria have an estimated sensitivity between 84–88% and an estimated specificity between 26–34% indicating that at least 1 in 10 septic patients are not captured using these criteria.^11^–^13^ The criteria are intended to be more sensitive than specific to ensure a diagnosis of sepsis due to the high mortality rates associated with delayed recognition and treatment.

**Population Health Research Capsule**

What do we already know about this issue?

*Sepsis is a life-threatening condition; systemic inflammatory response syndrome (SIRS) criteria is a diagnostic tool for sepsis*.

What was the research question?

*What is the effect of a modified SIRS criteria with addition of altered mental status and reduced heart rate for atrioventricular nodal blockers?*

What was the major finding of the study?

*Modified SIRS had an area under the curve (AUC) of 0.797 (95% CI, 0.78*–*0.809; P < .001), outperforming the original SIRS (AUC 0.7640.751–0.778; P < .001)*.

How does this improve population health?

*In elderly patients with sepsis, altered mental status may indicate end organ dysfunction, and atrioventricular nodal blockers may blunt tachycardia in the setting of infection*.

Atrioventricular nodal blocking medications such as beta-blockers and calcium channel blockers are commonly used as therapy to treat cardiovascular disease and dysrhythmias. Cardiovascular disease and dysrhythmias (ie, atrial fibrillation) are highly prevalent among patients ≥ 65 years. These medications may blunt tachycardia in patients with infection possibly leading to missed sepsis cases in this population. A reduced heart rate cutoff of 75 bpm for patients taking atrioventricular nodal blocking medications was chosen based on clinical experience and medical literature review.^14^–^17^ Multiple studies evaluating effects of atrioventricular nodal blockers, especially beta blockers, have demonstrated that an average heart rate reduction of 10–30 bpm (with a reduction of at least 15 bpm in several studies) translates into decreased mortality rates. Given this target for heart rate reduction when using these medications, in this study we used a reduced heart rate 15 bpm less than the current SIRS heart rate criterion of 90 bpm.^14^–^17^ Altered mental status is present in 20–70% of patients diagnosed with sepsis and was, therefore, chosen as an addition to the modified criteria.^18^,^19^ Our goal was to increase the sensitivity of SIRS criteria for patients ≥ 65 years of age by modifying the criteria to include a reduced heart rate for patients taking atrioventricular nodal blocking medications and altered mental status.

## METHODS

### Study Design and Setting

We conducted this retrospective observational cohort study at an 864-bed academic medical center in Lakeland, Florida. Inclusion criteria were adults ≥ 65 years of age who were admitted between January 1, 2022–August 31, 2023. We performed an a priori sample size calculation using G*Power (University of Düsseldorf, Germany) for binary logistic regression models using a calculated effect size (odds ratio [OR] 1.33), α = .05, with 80% power, and we determined that a minimum of 2,232 records were necessary to power the study. The institutional review board approved the study with a waiver of informed consent.

A total of 4,839 patients met inclusion criteria and were included in the analysis. We applied the abstraction standards of Worster et al, including patients selected through a query of electronic health records grouping patients into two approximately equal cohorts: 2,400 patients diagnosed with sepsis and 2,439 patients admitted for non-sepsis conditions serving as controls. Medical records identified were scrubbed of identifying data in an automated manner, although reviewers were not blinded to the hypothesis.^20^ The control group was selected from patients who were admitted to the hospital during the same date range but did not receive a sepsis diagnosis and were matched in a 1:1 ratio based on age, sex, race, and ethnicity with the experimental group with an equal number of patients taking atrioventricular nodal blocking medications. We excluded patients with missing data on sepsis status, altered mental status, or heart rate. Ages greater than 89 were masked as a binary variable per institutional data-protection policies. Patients were identified through a structured query by the institution’s analytics team.

### Sepsis Definition

Sepsis was identified using *International Classification of Diseases, 10^th^ Revision* (*ICD-10)* codes A41.9 (sepsis), R56.20 (severe sepsis), and R65.21 (septic shock), which were combined into a binary variable (sepsis vs non-sepsis).

### Systemic Inflammatory Respiratory Syndrome and Modified SIRS Criteria

The study compared traditional SIRS criteria to a modified version that incorporated two additional components: 1) altered mental status; and 2) heart rate > 75 bpm in patients prescribed atrioventricular nodal blocking agents (Table 1). A positive SIRS alert was defined as meeting two or more of the criteria. Altered mental status was determined using *ICD-10* codes for altered mental status (R41.82), altered level of consciousness (R41.0), and metabolic encephalopathy (G93.41). For patients on atrioventricular nodal blocking agents (eg, beta-blockers, calcium channel blockers), the modified SIRS criteria included those with a recorded heart rate > 75 bpm during hospitalization.

### Statistical Analysis

Our primary aim in the analysis was to evaluate how many additional sepsis patients were identified using the modified SIRS criteria compared to the original criteria and to assess the relative strength of each individual criterion in relation to sepsis diagnosis. We used descriptive statistics to quantify how many additional patients were captured by the modified criteria. Chi-square (χ^2^) tests were used to examine the bivariate association between each individual SIRS criterion and sepsis diagnosis. To assess the contribution of each individual criterion in the context of the others, we performed two binary logistic regression models. The first model included only the original four SIRS criteria, while the second model included the modified criteria— adding altered mental status and a heart rate > 75 bpm for patients on atrioventricular nodal blocking agents. In the modified SIRS regression model, we converted the original heart rate variable (> 90 bpm) only to patients not taking atrioventricular nodal blockers. These models were not intended to adjust for confounding variables. Rather, the purpose was to evaluate the association between each criterion and the likelihood of sepsis, considering the simultaneous presence of the other criteria, and the overall models’ sensitivity. Sensitivities and specificities for screening for and accurately being associated with sepsis were calculated for each individual criterion.

Model diagnostics included the Hosmer-Lemeshow goodness-of-fit test (with non-significant *P*-values indicating good model fit), Nagelkerke R^2^ to reflect variance explained, odds ratios, and 95% confidence intervals.^21^ All analyses were conducted using SPSS Statistics v29 (IBM Corporation, Armonk, NY). We used receiver operator characteristic (ROC) curve and area under the curve (AUC) to examine the sensitivity and specificity characteristics of each model.

## RESULTS

### Sample Characteristics

Among 4,839 patients included in the study, 51% were male, 87% were White, 92% were non-Hispanic, and 89% were 65–89 years of age. A total of 140 patients experienced a heart rate exceeding 170 bpm, of whom 116 (83%) were in the sepsis group. Most patients met both the original (n = 4,284; 88.5%) and modified SIRS criteria (n = 4,468; 92.3%) as described in Table 2. There were 184 patients who met the modified SIRS criteria but not the original SIRS criteria. Of these, 29 (15.8%) had a sepsis diagnosis, representing more than 1% of the total sepsis cohort. Of these 29 patients, 14 met the altered mental status criterion and 21 met the atrioventricular nodal blocker heart rate criterion, supporting their additive value.

### Chi-Square Analysis

We tested each criterion from the original and modified SIRS definitions using chi-square tests for association with a sepsis diagnosis (Table 3). The white blood cell (WBC) criterion was significantly associated with sepsis (χ^2^ = 858.99, *P* < .001), as was temperature (χ^2^ = 286.65, *P* < .001), respiratory rate (χ^2^ = 74.70, *P* < .001), and heart rate > 90 bpm (χ^2^ = 297.16, *P* < .001). Altered mental status, a component of the modified SIRS criteria, was also significantly associated with sepsis (χ^2^ = 464.37, P < .001). The heart rate > 75 bpm criterion among patients on AV nodal blockers, when examined alone, was not significantly associated with sepsis (χ^2^ = 1.78, P = .82).

### Sensitivity and Specificity of Individual Criteria

Individual criteria demonstrated wide variability in diagnostic performance with individual sensitivity and specificity (Table 4). Temperature abnormality demonstrated lower sensitivity at 29.1% (95% CI, 27.3–30.9%) but the highest specificity at 90.2% (88.9–91.3%). The respiratory rate criterion exhibited the highest sensitivity at 95.4% (94.5–96.2%) with low specificity at 11.3% (10.1–12.6%). Among patients on atrioventricular nodal blocking agents, the modified heart rate criterion (≥ 75 bpm) showed moderate sensitivity at 48.0% (46.0–50.0%) and moderate specificity at 53.9% (51.9–55.8%). Finally, altered mental status demonstrated lower sensitivity at 38.3% (36.4–40.3%), but high specificity at 88.4% (87.1–89.6%), reflecting its role as a more specific but less common indicator among septic patients.

### Logistic Regression Analysis

In the multivariable logistic regression analysis, all individual SIRS and modified SIRS criteria were independently associated with sepsis after adjusting for the presence of the other criteria as described in Table 5. The modified SIRS model, which incorporates altered mental status and stratified heart-rate criteria based on atrioventricular nodal blocker use, demonstrated improved explanatory power (Nagelkerke *R*^2^ = 0.355 vs 0.304) and a similarly good fit (Hosmer-Lemeshow *P* = .383). Within this expanded model, abnormal WBC count again remained the strongest predictor (OR 6.17, 95% CI 5.30–7.18, *P* < .001). Altered mental status also showed a robust association with sepsis (OR 3.95, 3.34–4.67, *P* < .001), as did heart rate ≥ 90 bpm among patients not on nodal blockers (OR 3.71, 2.75–4.99, *P* < .001) and the modified heart rate criterion of >75 bpm among atrioventricular nodal blocker users (OR 3.19, 2.37–4.30, *P* < .001). Together, these findings show that the modified model provides stronger overall discrimination and that both altered mental status and use of atrioventricular nodal blocker, and adjusted heart rate criterion contribute meaningful, independent predictive information beyond the traditional SIRS variables.

Statistics breakdown comparing average temperature, respiratory rate, heart rate, WBC, and lactic acid level with associated standard deviations and *P*-values for the sepsis group vs the control (non-sepsis) group are described in Table 6.

### Diagnostic Performance of Systemic Inflammatory Response Syndrome and Modified SIRS Criteria

The original SIRS criteria demonstrated a sensitivity of 97.7% (95% CI, 97.0–98.2%) and specificity of 20.5% (18.9–22.1%). The modified SIRS criteria improved sensitivity to 98.9% (98.4–99.2%), although specificity decreased to 14.1% (12.8–15.5%) as described in Table 7. The modified criteria identified an additional 29 sepsis cases not captured by the original SIRS criteria, representing 1.21% of the sepsis cohort (0.82–1.75%). Of these patients, 14 met the altered mental status criterion and 21 met the atrioventricular nodal-adjusted heart rate criterion.

### Receiver Operating Characteristic Curve and AUC

The modified model demonstrated higher sensitivity (84.5%) compared to the original model and better overall classification accuracy (71.9%; see Figure). The area under the ROC curve for the modified model demonstrated an AUC of 0.797 (95% CI, 0.785–0.809; *P* < .001), outperforming the original model (AUC 0.764; 0.751–0.778; *P* < .001). Sensitivity remained high across practical probability thresholds.

## DISCUSSION

Overall, when comparing the application of the current SIRS criteria with the modified SIRS criteria for the sampled patients ≥ 65 years of age who were diagnosed with sepsis, the current SIRS criteria accurately captured 97.7% (95% CI, 97.0–98.2%) of septic patients and the modified SIRS criteria accurately captured 98.9% (98.4–99.2%) of septic patients. While the modified criteria had a higher sensitivity, the specificity for the modified criteria (14.1%, 12.8–15.5%) was lower compared to the original (20.5%, 18.9–22.1%) as described in Table 2. These criteria are meant to be highly sensitive and, therefore, may lack specificity in an attempt to capture all possible septic patients. Many non-infectious causes can meet SIRS criteria such as trauma, burns, uncontrolled pain, etc; therefore, clinical judgment and identification of an infectious source must be made to confirm a diagnosis of sepsis. While the modified SIRS criteria accurately captured only 1.2% higher number of septic patients than the current SIRS criteria, this represents 1 in every 100 patients ≥ 65 years of age diagnosed with sepsis, which is a large population considering sepsis affects tens of millions of patients each year.^2^,^3^

Using a regression analysis, it was determined that white blood cell count criteria was the most highly associated criterion with sepsis (OR 6.17), and altered mental status criterion was the second (OR 3.95) as described in Table 5. Altered mental status was associated with a true sepsis diagnosis in 76.5% (95% CI, 74.1–78.8%) of patients (Table 3). Altered mental status is a common finding in sepsis and is an indicator of end-organ damage and increased mortality as described in the quick Sequential Organ Failure Assessment (qSOFA).^22^ Disturbances in the blood brain barrier allow for the passage of neurotoxic factors caused by infection, resulting in toxic metabolic encephalopathy. Hypoxemia, ischemia, acidemia, oxidative stress, and inflammatory mediators also contribute to encephalopathy.^23^ Clinicians should recognize altered mental status as an indicator of end-organ dysfunction even if a patient has reassuring vital signs and unremarkable labs (ie, no leukocytosis, normal lactic acid level), possibly indicating sepsis when an infection is identified or suspected.

While altered mental status is used in other tools for identifying sepsis and stratifying its severity such as in the qSOFA criteria, no literature until now has suggested the use of a reduced heart rate criterion for patients taking atrioventricular nodal blocking medications. This new criterion for a reduced heart rate affects a large population of the targeted age group of patients ≥ 65 years of age. Nearly half of the sampled population were taking an atrioventricular nodal blocking medication (2,348 of the total sample; 1,164 patients in the septic cohort), and an additional 7% (83 patients) diagnosed with sepsis and taking an atrioventricular nodal blocker were captured by the new criteria of a reduced heart rate of 75 bpm (Table 2). If this were extrapolated to the general population then this new criterion would apply to nearly half of all patients ≥ 65 years of age. It should also be noted that the average heart rate for these patients as described in Table 6 may be skewed due to dysrhythmias. There were 140 patients (116 in the sepsis cohort) with a heart rate over 170 bpm, which was supraphysiologic for their age. These patients likely experienced a dysrhythmia such as atrial fibrillation/flutter with rapid ventricular response, supraventricular tachycardia, ventricular fibrillation, or ventricular tachycardia.

Temperature was the most specific criterion for diagnosing sepsis with a specificity of 90.2% (95% CI, 88.9–91.3%) followed by altered mental status (88.4%; 87.1–89.6%) and then heart rate > 75 bpm among patients who used atrioventricular nodal blockers (53.9%; 51.9–55.8%), as described in Table 4. This demonstrates that while the overall modified SIRS criteria had a lower specificity, likely due to more variables being included, the new criteria of altered mental status and reduced heart rate for patients on atrioventricular nodal blocking medications were highly specific independently for sepsis.

The use of modified SIRS criteria that includes altered mental status and a decreased heart rate cutoff for patients using atrioventricular nodal blocking medication has the potential benefit of facilitating a diagnosis of sepsis earlier in a vulnerable elderly population. The proposed modified SIRS criteria had a higher sensitivity although specificity was lower, and the model fit was also improved with a higher sensitivity than the original SIRS when evaluating the ROC and AUC. The modified criteria have the potential to be especially useful in the prehospital setting, as well as when patients are determined to be “sepsis alerts” prior to arrival to the hospital. The SIRS criteria are commonly used by emergency medical services (EMS) to determine whether a patient qualifies as a “sepsis alert,” since first responders do not have the ability to access lab results (eg, WBC or lactic acid) to make this determination. Including a lower heart rate cutoff for patients taking beta-blockers or non-dihydropyridine calcium channel blockers and an altered mental status may allow for increased recognition of possible septic patients in the prehospital setting by EMS and allow for expedited care and treatment.

While the Third International Consensus Definitions for Sepsis and Septic Shock (Sepsis-3) have moved away from SIRS criteria to focus more on qSOFA, this is not a useful screening tool in the ED and has been validated only to predict mortality.^1^ The SIRS criteria are still widely used as the primary screening tool for sepsis in the ED due to its high sensitivity and inclusion in hospital sepsis metrics. The SIRS criteria depend heavily on patient vital signs; however, in this study we have shown that depending on an elevated heart rate as a tool for clinicians to guide whether a patient may be septic is not always reliable. This is especially important in patients over > 65 years of age taking atrioventricular node blocking medications, as this study demonstrated that a heart rate of only 75 bpm or higher in these patients was highly associated with sepsis. These patients should have special consideration when being evaluated as they may be more ill than their seemingly reassuring vital signs may indicate.

## LIMITATIONS

Given the retrospective nature of this study, it inherently has limitations and is also limited by being a single-center study. The standard for diagnosing sepsis in the emergency setting is the current SIRS criteria; thus, in a retrospective observational study using this standard, it would be expected that many positive sepsis diagnoses meet the SIRS criteria. Patients were determined to have sepsis if they had an admitting diagnosis of sepsis; however, some patients may have met sepsis criteria but did not receive a formal diagnosis. Similarly, altered mental status was determined in this study if patients had a concurrent admitting diagnosis for the condition; however, it is possible that patients may have presented altered but did not receive a formal diagnosis code and, therefore, would have been captured by the altered mental status criterion but were not in this study. The use of *ICD-10* codes may be associated with problems such as inaccurate coding, possibly leading to under-reporting or over-reporting of conditions. Additional limitations include not knowing whether patients were in fact in compliance with their atrioventricular nodal blocking medications. Neither was it clear how many patients met SIRS criteria or lack thereof due to comorbid diagnoses (ie, chronic heart failure, dehydration, gastrointestinal bleeding, etc) as opposed to sepsis.

Further studies with additional measures for determining true sepsis diagnoses such as trending lactic acid levels, creatinine levels for acute kidney injury, or other indicators of end-organ damage are warranted to compare the current SIRS criteria and newly proposed modified SIRS criteria to validate or disprove their use in patients > 65 years of age. Additional studies using data from multiple healthcare centers, such as mining larger datasets using software like COSMOS (Epic Systems Corporation, Verona,WI) is also warranted to further evaluate the use of this modified SIRS criteria.

## CONCLUSION

This study demonstrates that the proposed modified SIRS criteria with the addition of altered mental status criterion and a reduced heart rate criterion for patients taking atrioventricular blocking medications for patients > 65 years of age are associated with a higher sensitivity for sepsis than the current SIRS criteria, although specificity decreased. When evaluating a criterion’s specificity for sepsis, altered mental status and reduced heart rate of > 75 bpm among patients using atrioventricular nodal blocking medications were the second and third most specific individual criterion, respectively. While the proposed modified SIRS criteria may result in higher rates of false-positive screenings with a lower specificity, it also may identify higher rates of septic patients in a vulnerable elderly population, thereby identifying an additional 1.21 in every 100 septic patients in this age group.

Future prospective and multicenter studies that better control for confounding effects and evaluate downstream clinical impacts are warranted. Future prospective studies may benefit from additional measures for determining true sepsis diagnoses such as trending lactic acid levels, creatinine levels, or other indicators of end-organ damage to compare the current SIRS criteria and newly proposed modified SIRS criteria to validate or disprove its use in patients > 65 years of age.

## Figures and Tables

**Figure:**
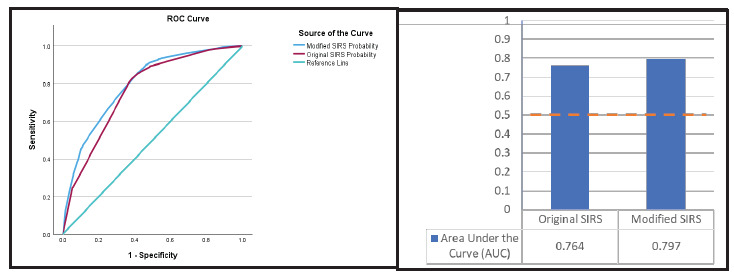
Receiver operating characteristic curve and area under the curve comparison. ROC curve and AUC comparing model fit of original vs modified systemic inflammatory response syndrome criteria. Modified SIRS had a larger AUC indicating a better model. Note: A good model has a value > 0.5. A value = 0.5 indicates the model is no better than random prediction. *AUC*, area under the curve; *ROC*, receiver operating characteristic; *SIRS*, systemic inflammatory response syndrome.

**Table 1:** Comparison of original severe inflammatory response (SIRS) criteria and modified SIRS criteria for patients ≥ 65 years of age.

Original SIRS Criteria	Modified SIRS Criteria for Patients ≥ 65
WBC < 4 k or > 12,000 cells/mm^3^, or > 10% immature bands	WBC < 4,000 or > 12,000 cells/mm^3^, or > 10% immature bands
Heart rate > 90 bpm	Heart rate > 90 bpm
Tachypnea > 20 breaths/min or PaCO2 < 32 mm Hg	Tachypnea > 20 breaths/min or PaCO_2_ < 32 mm Hg
Temperature < 36 ºC or >38 ºC	Temperature < 36 ºC or >38 ºC
	**Heart rate > 75 bpm for patients taking atrioventricular nodal blocking medications**
	**Altered Mental Status**

Each criterion is assigned 1 point; 2 points indicate a positive screening. Positive screening with an identified source of infection meets sepsis criteria.

*bpm*, beats per minute; *mm Hg*, millimeters of mercury; *mm^3^*, cubic millimeter; PaCO_2_; partial pressure of arterial carbon dioxide; *SIRS*, systemic inflammatory response syndrome; *WBC*, white blood cell.

**Table 2:** Sample description with all criteria in a study comparing systemic inflammatory response syndrome (SIRS) with modified SIRS critiera.

	n (%) of Patients with Sepsis	n (%) of Patients without Sepsis	N (%) of Total Cohort
Total Sample %	2,400 (49.3)	2,439 (50.4)	4,839 (100)
Sex			
Female	1,125 (46.9)	1,227 (50.3)	2,352 (48.6)
Male	1,275 (53.1)	1,212 (49.7)	2,487 (51.4)
Age			
65–89	2,170 (90.4)	2,164 (88.7)	4,334 (89.6)
> 89	230 (9.6)	275 (11.3)	505 (10.4)
Race			
Asian	(< 1)	(< 1)	38 (0.8)
Black	227 (9.5)	284 (11.6)	511 (10.6)
Pacific Islander/ Native American/ Alaskan	(< 1)	(< 1)	8 (0.2)
Unknown	38 (1.6)	23 (< 1)	61 (1.3)
White	2,107 (87.8)	2,114 (86.7)	4,221 (87.2)
Ethnicity			
Hispanic	158 (6.6)	160 (6.6)	318 (6.6)
Non-Hispanic	2,189 (91.2)	2,256 (92.5)	4,445 (91.9)
Unknown	53 (2.2)	23 (1.0)	76 (1.6)
On AV nodal blockers	1,164 (48.5)	1,184 (48.5)	2,348 (48.5)
WBC criteria	2,078 (86.6)	1,142 (46.8)	3,220 (66.5)
Respiratory criteria	2,290 (95.4)	2,163 (88.7)	4,453 (92.0)
Temperature criteria	698 (29.1)	240 (9.8)	938 (19.4)
HR > 90 bpm (original criteria)	2,245 (93.5)	1,844 (75.6)	4,089 (84.5)
Not on AV nodal blockers HR > 90 bpm	1,175 (49.0)	964 (39.5)	2,139 (44.2)
On AV nodal blockers and HR > 75 bpm	1,153 (48.0)	1,125 (46.1)	2,278 (47.1)
On AV nodal blockers and HR > 75 but < 90 bpm	83 (3.5)	245 (10)	328 (6.8)
Altered mental status	920 (38.3)	282 (11.6)	1,202 (24.8)
Original SIRS criteria	2,344 (97.7)	1,940 (79.5)	4,284 (88.5)
Modified SIRS criteria	2,373 (98.9)	2,095 (85.9)	4,468 (92.3)

Sample description of patients included with sepsis and non-sepsis diagnoses. Criteria met for original and modified SIRS criteria are included in descriptors. Sensitivity for the original criteria was 97.7% and 98.9% for the modified criteria.

*AV*, atrioventricular; *bpm*, beats per minute; *HR*, heart rate; *SIRS*, systemic inflammatory response syndrome; *WBC*, white blood cell.

**Table 3:** Individual criterion breakdown from chi-square analysis among sampled population.

		No Sepsis (control)	Sepsis	Total
White Blood Cell Criterion (P < .001)				

Did not meet temperature criterion	Count	2,199	1,702	3,901
	% within temp criterion	56.4%	43.6%	100.0%
Met temperature criterion	Count	240	698	938
	% within temp criterion	25.6%	74.4%	100.0%
Total	Count	2,439	2,400	4,839
	% within temp criterion	50.4%	49.6%	100.0%

Respiratory Rate Criterion (P < .001)				

Did not meet respiratory criterion	Count	276	110	386
	% within respiratory criterion	71.5%	28.5%	100.0%
Met respiratory criterion (tachypnea)	Count	2,163	2,290	4,453
	% within respiratory criterion	48.6%	51.4%	100.0%
Total	Count	2,439	2,400	4,839
	% within respiratory criterion	50.4%	49.6%	100.0%

Heart Rate Original Criterion ≥ 90 bpm (P < .001)				

Did not meet original HR criterion	Count	595	155	750
	% within HR original criterion	79.3%	20.7%	100.0%
Met original HR criterion	Count	1,844	2,245	4,089
	% within HR original criterion	45.1%	54.9%	100.0%
Total	Count	2439	2,400	4,839
	% within HR original criterion	50.4%	49.6%	100.0%

Atrioventricular Nodal Blocker Heart Rate Criterion ≥ 75 bpm (P = 0.18)				

	Count	1,314	1,247	2,561
Did not meet AV nodal blocker HR ≥ 75 bpm criterion	% within HR Crit75NB	51.3%	48.7%	100.0%
	Count	1,125	1,153	2,278
Met AV nodal blocker HR ≥ 75 bpm criterion	% within HR Crit75NB	49.4%	50.6%	100.0%
	Count	2,439	2,400	4,839
Total	% within HR Crit75NB	50.4%	49.6%	100.0%

Altered Mental Status Criteria (P < .001)				

Did not meet altered mental status (AMS) criterion	Count	2,157	1,480	3,637
	% within AMS	59.3%	40.7%	100.0%
Met AMS criterion	Count	282	920	1,202
	% within AMS	23.5%	76.5%	100.0%
Total	Count	2,439	2,400	4,839
	% within AMS	50.4%	49.6%	100.0%

Criteria breakdown from chi-square analysis among sampled population. Each criterion demonstrated a significant association with sepsis (P < .001), except for the AV nodal blocker heart rate criteria ≥ 75 bpm, which had a P-value = .18. Altered mental status had the strongest association with sepsis (76.5%), followed by the temperature criterion (74.4%).

*AV*, atrioventricular; *HR*, heart rate; *NB*, nodal blocker; *WBC*, white blood cell.

**Table 4:** Sensitivity and specificity of individual systemic inflammatory response syndrome (SIRS) criteria and modified SIRS criteria.

Criterion	Sensitivity	95% CI	Specificity	95% CI
Respiratory rate	95.4%	94.5–96.2%	11.3%	10.1–12.6%
Heart rate ≥ 90 bpm	93.5%	92.5–94.5%	24.4%	22.7–26.1%
WBC	86.6%	85.2–87.9%	53.2%	51.2–55.2%
HR ≥ 75 bpm among AV nodal blocker users	48.0%	46.0–50.0%	53.9%	51.9–55.8%
Altered mental status	38.3%	36.4–40.3%	88.4%	87.1–89.6%
Temperature	29.1%	27.3–30.9%	90.2%	88.9–91.3%

Sensitivity and specificity of individual criteria for original SIRS and modified SIRS criteria including 95% CI. Criteria are listed in descending order from highest sensitivity to lowest. Temperature was the most specific criterion for diagnosing sepsis followed by altered mental status and then heart rate > 75 bpm among AV nodal blocker users. Respiratory rate was the most sensitive criterion for screening for sepsis followed by heart rate > 90 bpm and then white blood cell count.

*AV*, atrioventricular; *bpm*, beats per minute; *HR*, heart rate; *SIRS*, systemic inflammatory response syndrome; *WBC*, white blood cell.

**Table 5:** Regression model breakdown comparison.

	Significance	Odds Ratio	95% CI (lower)	95% CI (upper)
Original SIRS Criteria Regression Model				
WBC criteria	<.001	6.16	5.31	7.13
Temperature criteria	<.001	2.86	2.40	3.40
Respiratory rate criteria	<.001	1.57	1.21	2.03
HR original (≥ 90)	<.001	3.16	2.57	3.87
Modified SIRS Criteria Regression Model				
WBC criteria	<.001	6.17	5.30	7.18
Temperature criteria	<.001	2.44	2.04	2.92
Respiratory rate criteria	.003	1.48	1.14	1.92
HR original (≥ 90), no NB	<.001	3.71	2.75	4.99
HR modified (≥ 75), + NB	<.001	3.19	2.37	4.30
Altered Mental Status	<.001	3.95	3.34	4.67

Odds ratio was used to determine each individual criterion’s association with a positive sepsis diagnosis. WBC criteria had the highest odds ratio association with sepsis when using the modified criteria with OR of 6.17 followed by altered mental status (OR 3.95) and HR ≥ 90 (OR 3.71).

*HR*, heart rate; *AV*, atrioventricular nodal blocking medication; *NB*, nodal blocker; *OR*, odds ratio; *SIRS*, systemic inflammatory response syndrome; *WBC*, white blood cell.

**Table 6:** Comparison of vital signs, white blood cell count, and lactic acid level between the sepsis and the control (non-sepsis) cohort.

		N	Mean	SD	P-value
Average Temperature	Sepsis	2,400	36.94	.622	< .001
	Control	2,439	36.77	.320	< .001
Average Respiratory Rate	Sepsis	2,400	21.24	5.228	< .001
	Control	2,439	19.25	4.114	< .001
Average Heart Rate	Sepsis	2,400	92.48	15.125	< .001
	Control	2,439	83.56	14.018	< .001
Average WBC	Sepsis	2,400	14.63	8.378	< .001
	Control	2,439	9.31	6.369	< .001
Average Lactic Acid Level	Sepsis	2,277	2.89	2.177	< .001
	Control	1,097	1.82	1.387	< .001

*WBC*, white blood cell.

**Table 7:** Sensitivity and specificity of original systemic inflammatory response syndrome (SIRS) vs modified SIRS criteria.

	Original SIRS Criteria		Modified SIRS Criteria	
	
	Sepsis Diagnosis	No Sepsis Diagnosis	Sepsis Diagnosis	No Sepsis Diagnosis
Positive screening	2,344	1,940	2,373	2,095
Negative screening	56	499	27	344
	Sensitivity = 97.7% (95% CI, 97.0–98.2%)	Specificity = 20.5% (95% CI, 18.9–22.1%)	Sensitivity = 98.9% (95% CI, 98.4–99.2%)	Specificity = 14.1% (95% CI, 12.8–15.5%)

*SIRS*, systemic inflammatory response syndrome.
